# Five-step unilateral biportal endoscopic surgery for central lumbar canal stenosis: "Z" technique nuance

**DOI:** 10.3171/2024.1.FOCVID23182

**Published:** 2024-04-01

**Authors:** Ariel Kaen, Javier Quillo-Olvera, Man Kyu Park, Santiago Rocha, Fernando Durand, Ignacio Martin, Sang-Kyu Son

**Affiliations:** 1 Department of Neurosurgery, Hospital Virgen del Rocío, Seville, Spain;; 2 Neurosurgery Unit, Spine Center, Hospital Angeles Centro Sur, Querétaro City, Mexico;; 3 Department of Neurosurgery, Good Gangan Hospital, Busan, Korea; and; 4 Department of Neurosurgery, Good Moonhwa Hospital, Busan, Korea

**Keywords:** spinal stenosis, endoscopic, decompression, lumbar, unilateral biportal endoscope, UBE

## Abstract

The treatment for lumbar spinal stenosis has advanced through the use of minimally invasive surgery techniques. Endoscopic methods go even further, with studies showing that both uniportal and biportal endoscopic techniques have outcomes comparable to traditional approaches. However, there is limited knowledge of the step-by-step decompression process when using the unilateral biportal endoscopic (UBE). To address this, the authors introduce the five steps in the "Z" sequence, which aims to reduce surgical time and complications.

The video can be found here: https://stream.cadmore.media/r10.3171/2024.1.FOCVID23182

## Transcript

Five-step unilateral biportal endoscopic surgery for central lumbar canal stenosis: "Z" technique nuance.

Lumbar central canal stenosis is a spinal degenerative disease common in elderly patients. Biportal endoscopic spine surgery has become a popular alternative option for treatment. Here we present a five-step biportal endoscopic approach.

### 0:47

General or epidural anesthesia can be used in endoscopic spine surgery. On a true anteroposterior view, two vertical lines should be drawn, one at the midline and the other at the medial pedicle line. The target point is the spinous laminar junction, your instruments must be oriented to this point. The caudal incision should be made at the medial border of the pedicle, and the cranial incision should be slightly lower than the pedicle.

### 1:15

Serial dilators should be inserted and touch the spinous lamina junctions. Continuous fluent irrigation from the endoscopic portal to the working portal helps you to maintain a clean operative surgical view.

### 1:27

There are two different sequences described using endoscopic decompression. We will refer to the most commonly used technique as the "N" technique, which follows a craniocaudal order. The first step involves drilling the lower edge of the upper vertebra until the flavum ligament is completely detached. The second step focuses on decompressing the ligament at its caudal insertions. Step 3 begins contralateral laminotomy using the "over-the-top" technique. The final step involves detaching the contralateral ligament at the upper edge of the lower vertebra.

### 2:02

In this video we will be discussing a technique called the "Z" technique for laminectomy in a "side-to-side" direction. This technique starts with drilling the lower edge of the upper vertebra and then moving the instruments contralaterally to detach the flavum ligament along its entire upper edge. In step 3, start again at the upper border of the lower vertebra and decompression is completed when the contralateral flavum ligament is detachment.

### 2:29

The "Z" technique enables the authors to use endoscopic instruments more efficiently. In the cranial portion we primarily use the shaver or drill along with the Kerrison punch, while in the caudal half the osteotome and rotating Kerrison punch are more useful.

### 2:46

The first step begins with the ipsilateral laminotomy. This is a critical step that involves drilling the lower edge of the upper vertebra until the upper limit of the flavum ligament is visible. It is essential to note that this drilling not only detaches the flavum ligament, but also creates space for the endoscope camera in the upcoming steps.

### 3:06

In this first video, a left biportal endoscopic approach is performed. Remember that always the left side of the screen is cranial and the upper part of the video is medial.

In the first step, the goal is to detach the flavum ligament from the superior insertion.

You should remove bone from the inferior edge of the upper laminar with a drill or Kerrison punch. This second video shows the final part of the first step when we can identify the cranial epidural space.

### 3:34

In addition to drilling the lamina, step 1 also involves drilling the base of the spinous process. The flavum ligament is shaped like a butterfly and has a significant anatomical landmark known as the "midline cleft," where it meets the ligament on the opposite side. This cleft serves as a crucial landmark for identifying the midline location.

In these two videos you can see how the midline cleft of the flavum ligament guides the surgeon in the location of the midline. This anatomical landmark is present even in patients with severe degenerative lumbar change.

### 4:09

To apply the "Z" technique, the next step is step 2, which involves contralateral decompression of the upper part on the opposite lamina. To conduct it you should move to the opposite side of the neural elements by passing "over the top" of them, which is known as unilateral laminotomy for bilateral decompression. It’s crucial to comprehend the location of the flavum ligament insertion while drilling the lamina on the opposite side.

### 4:31

The flavum ligament has two layers, the superficial and the deep. In the illustration, we can understand how the deep layer at its upper edge is inserted under the laminar of the upper vertebra, while the superficial layer is inserted at the free edge of that same lamina. With a star, we show the spinous lamina "inner point," the anatomical landmark where these two layers attach. Cranial to this point, the deep layer can easily be dissected in a caudal-cranial direction. Then with a caudal movement of a dissector, we can divide the deep layer from the superficial one. It is very useful to use the RF probe to remove the superficial layer of the ligamentum flavum in order to increase the working space.

### 5:16

In this endoscopic view during surgery, we are highlighting that inner spinous lamina point, and demonstrating how the two layers of the flavum ligament can be separated from the opposite side. It is crucial to preserve the deep layer to minimize the risk of dural tears and epidural bleeding. Again, we dissect the deep layer from caudal to cranial until observe complete detachment. Then with the RF probe, we removed the superficial layer. Now that we have completely dissected the flavum ligament, we can drill in an undercutting fashion on the opposite lamina. The deep layer acts as a protective barrier during drilling to minimize neural damage.

### 6:10

Step 3 consists of removing the ipsilateral superficial layer and detaching the deep layer at the lower attachments. To understand, we need to look back at the previous illustration. The superficial layer of the flavum ligament passes over the upper edge of the lower lamina. Using a curette that touches the bone, we can separate the superficial layer and remove the ligament in a downward to upward motion. Once again, it is essential to preserve the deep layer to prevent complications. The deep layer can be detached by rotating the Kerrison punch or by chisel.

### 6:51

This video demonstrates how to reduce the thickness of the flavum ligament by dissecting it from caudal to cranial. Finally, with the Kerrison punch, we can detach the deep layer from the ipsilateral lower attachment.

### 7:25

Step 4. Detachment of the inferior insertions of the contralateral flavum ligament. To detach the contralateral flavum ligament you have two options, using a Kerrison punch or an osteotome. If you prefer the Kerrison punch, begin at the junction of the lamina and the base of the superior articular process, as demonstrated in the first video.

Alternatively, you can use an osteotome to remove all the inferior insertions in one block.

### 8:00

Step 5. In the last step, only the flavum ligament is inserted in its most lateral portion, that is, the medial edge of the superior articular process. It is recommended to begin with the disinsertion of the contralateral side in order to use the ligament as a dura mater protector. Kerrison punch or osteotome can be used. As seen in this video, with the Kerrison punch a small bone notch is made at the base of the superior articular process. This makes it easier for the osteotome to not slide inadvertently, and make a clean cut at lateral the insertion of the ligament.

### 8:38

Then we can remove the flavum ligament from the ipsilateral side. In order to reduce facet joint damage it’s recommended to use a special instrument like a curve osteotome or curve Kerrison punch. As we demonstrated in a previous video, we can employ the same technical approach to achieve the desired results. First, we create a bone notch at the base of the ipsilateral superior articular process. Then using a curved osteotome we can fully detach the flavum ligament.

### 9:10

Finally, it is important to inspect the dural attachment below the ligament before removing the ligament as in a block. Reducing the punching may reduce the risk of dural tears. It is mandatory to check the complete decompression for both sides. As we see in this case, all the lateral recess of the contralateral side is decompressed. In this second case we show the ipsilateral decompression all the transversing nerve root free after biportal endoscopic surgery. On the right side of the video, the postoperative MRI shows the lumbar decompression correctly on sagittal and axial views.

### 9:53 Discussion.

Learning to perform MIS surgeries takes time and requires practice. Several studies have demonstrated that achieving the same results as open surgery requires between 20–30 cases. The learning curve for these procedures is relatively short and can be made even shorter if a step-by-step system is followed.

### 10:12 Complications.

Dural tear remained a major complication, with an incidence of 4.5%. Incomplete decompression was 2.0%, transient palsy was 2.6%, and symptomatic postoperative spinal epidural hematoma was only 1.1%

### 10:30 Conclusions.

This video has demonstrated UBE laminectomy with the "Z" technique has had safe and reproducible results to decompress lumbar central canal stenosis.^1–11^

## Disclosures

The authors report no conflict of interest concerning the materials or methods used in this study or the findings specified in this publication.

## Author Contributions

Primary surgeon: Kaen, Son. Assistant surgeon: Rocha. Editing and drafting the video and abstract: Kaen. Critically revising the work: Kaen, Quillo-Olvera, Park. Reviewed submitted version of the work: Kaen, Quillo-Olvera, Park. Approved the final version of the work on behalf of all authors: Kaen. Supervision: Kaen, Quillo-Olvera, Park, Durand, Martin, Son.

## Supplemental Information

Supplementary Figs. 1–3

### Patient Informed Consent

The necessary patient informed consent was obtained in this study.
