# Supplementary material for: Five-step unilateral biportal endoscopic surgery for central lumbar canal stenosis: "Z" technique nuance
Source: Neurosurg Focus Video. 2024 Apr 1;10(2):V3. doi: 10.3171/2024.1.FOCVID23182 (PMC11013373; doi:10.3171/2024.1.FOCVID23182)
Supplement: Supplementary Figs. 1–3 [file SupplementaryFigs1-3_FOCVID23-182.pdf]

NEUROSURGICAL
FOCUS
VIDEO

ONLINE ONLY

Supplemental material

Five-step unilateral biportal endoscopic surgery for central lumbar canal stenosis: "Z" technique nuance

Kaen et al.

https://thejns.org/doi/abs/10.3171/2024.1.FOCVID23182

DISCLAIMER The *Journal of Neurosurgery* acknowledges that the following section is published verbatim as submitted by the authors and did not go through either the *Journal's* peer-review or editing process.

© 2024 The authors, CC BY 4.0 (http://creativecommons.org/licenses/by/4.0/)

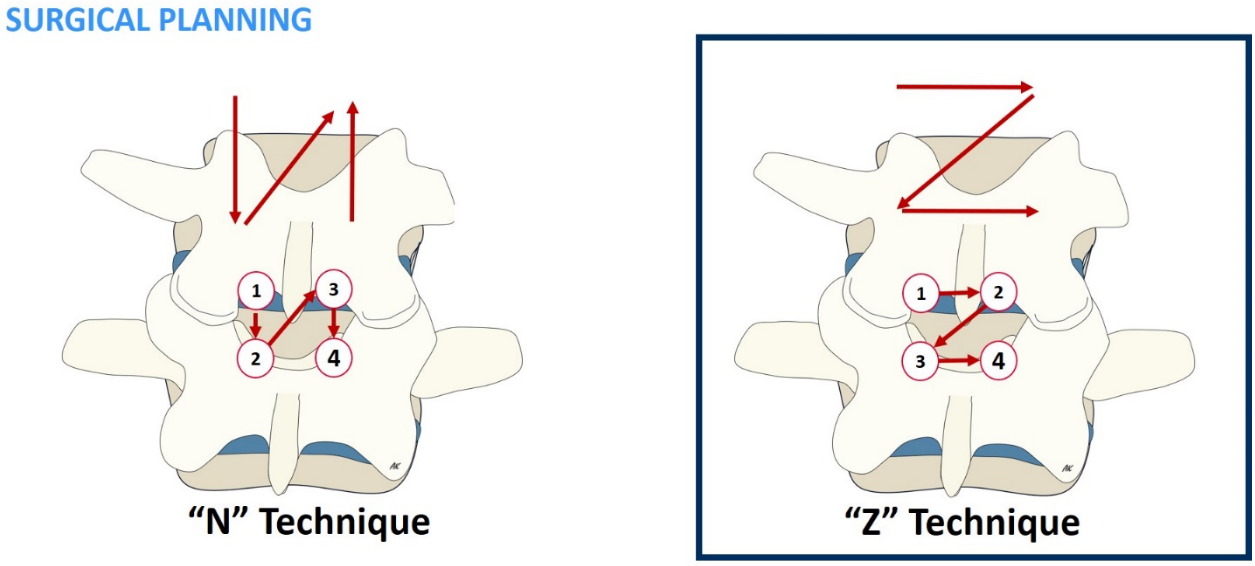

Supplementary Figure 1.

In these illustrations we show the sequential steps and the different between the classical "N" technique (right) and the new one "Z" technique (left).

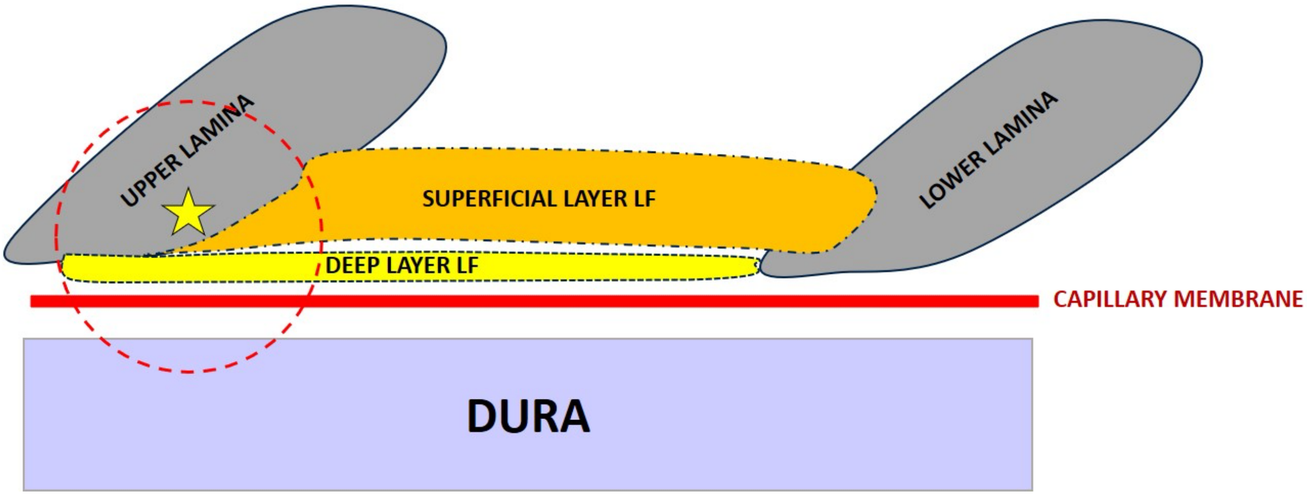

Supplementary Figure 2.

Illustrations that show the different insertions of the two layers of the flavum ligament.

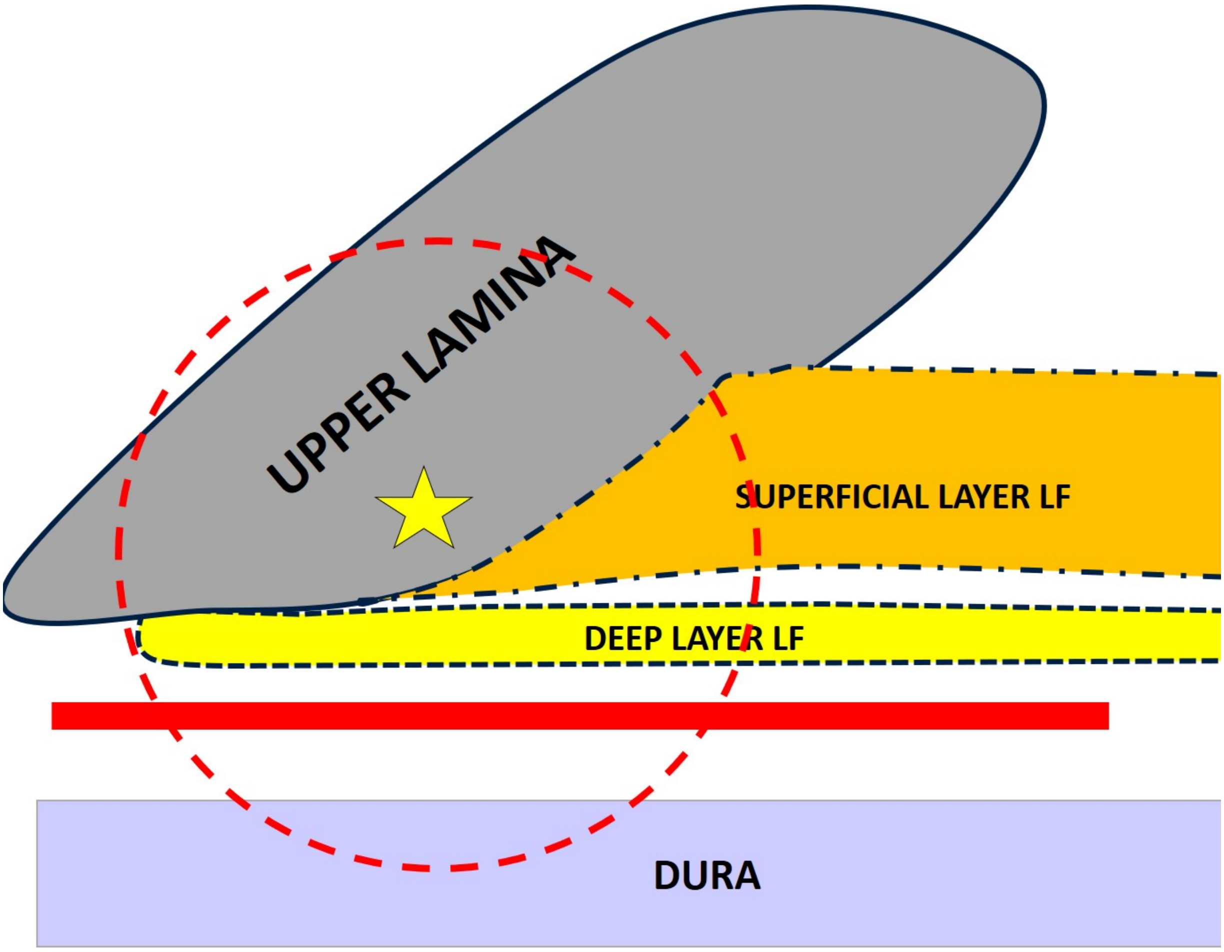

Supplementary Figure 3.

Illustrations that show the "inner" spinous laminar point (star), where is easier to dissect the superficial from the deeper layer of flavum ligament.
